# Quantum key distribution with hacking countermeasures and long term field trial

**DOI:** 10.1038/s41598-017-01884-0

**Published:** 2017-05-16

**Authors:** A. R. Dixon, J. F. Dynes, M. Lucamarini, B. Fröhlich, A. W. Sharpe, A. Plews, W. Tam, Z. L. Yuan, Y. Tanizawa, H. Sato, S. Kawamura, M. Fujiwara, M. Sasaki, A. J. Shields

**Affiliations:** 1 Toshiba Corporate Research & Development Center, 1 Komukai-Toshiba-Cho, Saiwai-ku, Kawasaki 212-8582 Japan; 2 Toshiba Research Europe Ltd, 208 Cambridge Science Park, Cambridge, CB4 0GZ UK; 3 Quantum ICT Laboratory, National Institute of Information and Communications Technology, 4-2-1 Koganei-city, Tokyo, 184-8795 Japan

## Abstract

Quantum key distribution’s (QKD’s) central and unique claim is information theoretic security. However there is an increasing understanding that the security of a QKD system relies not only on theoretical security proofs, but also on how closely the physical system matches the theoretical models and prevents attacks due to discrepancies. These side channel or hacking attacks exploit physical devices which do not necessarily behave precisely as the theory expects. As such there is a need for QKD systems to be demonstrated to provide security both in the theoretical and physical implementation. We report here a QKD system designed with this goal in mind, providing a more resilient target against possible hacking attacks including Trojan horse, detector blinding, phase randomisation and photon number splitting attacks. The QKD system was installed into a 45 km link of a metropolitan telecom network for a 2.5 month period, during which time the system operated continuously and distributed 1.33 Tbits of secure key data with a stable secure key rate over 200 kbit/s. In addition security is demonstrated against coherent attacks that are more general than the collective class of attacks usually considered.

## Introduction

Quantum Key Distribution^1^ (QKD) is well known for its unique information theoretic security, which does not depend on the resources available to an eavesdropper. In recent years experiments have demonstrated high rates of key distribution^2–4^ combined with network architectures^5, 6^ and standard data signals via multiplexing^7–10^. Progress is also being made on long term operation, deployment in real telecom networks and linking together multiple different QKD systems^11–14^.

As the experimental maturity of QKD has advanced so too has the understanding of important differences between the security assumptions of the theory and the physical implementation. These differences could potentially be exploited by an eavesdropper, allowing quantum hacking attacks which bypass the presumed quantum-enabled security. An early example of this was seen in the first QKD experiment^15^, when the audible movement of components leaked key information to anyone within hearing distance^16^. This constitutes a clear example of a “side channel” – a physical channel that is informative to the eavesdropper but is not included in the theoretical model. The presence of side channels is a problem facing all cryptographic devices. Classical cryptography hardware implementations have been demonstrated to be vulnerable to hacking targeting unexpected physical channels such as power usage^17^ or computation time^18^ instead of attacking the underlying mathematical algorithms.

In QKD, a number of different attacks have been proposed which exploit side channels in various different protocols. For the one way BB84 protocol the main examples of these attacks, and some typical countermeasures, are listed in Table 1. For other protocols see Table 1 in ref. 19. Countermeasures should ideally be connected to the system security proof via testable assumptions – this is done for example with decoy states, phase randomisation characterisation and also recently for Trojan horse optical components^20, 21^.

**Table 1:** Examples of side channel attacks on one way BB84 QKD.

Attack name	Target	Countermeasures
Photon number splitting^65^	Source	Decoy states^56, 57^, SARG04^66^
Trojan horse^52, 67^	Source/Receiver	Passive optical components^20, 52^
Phase randomisation^68^	Source	Active randomisation^69^, Characterisation^47^
Blinding^22^	Detector	MDI-QKD^35, 36^, Optical monitoring^28^, Detector monitoring^70^
Time shift^24, 29, 71^	Detector	MDI-QKD^35, 36^, Detector symmetrisation^72^
Dead-time^73^	Detector	MDI-QKD^35, 36^, Simultaneous dead-time^74^

Some of these attacks are well-known; for example the photon number splitting attack (which can be mitigated using the decoy state protocol) and detector control attacks^22–25^. Many of these attacks have also been demonstrated experimentally^26–30^. However, it is worth clarifying that reports of attacks breaking the security of QKD invariably refer to breaking a particular protocol and hardware implementation rather than breaking QKD in general. And it also should be said that attacks are typically not implementable in real world conditions, requiring theoretical technology or access to characterise the particular QKD units under attack. Nevertheless for robust security guarantees all information which can leak through side channel attacks for a given implementation should be bounded and removed through privacy amplification.

One possible way to remove side-channel information is to reduce the theory assumptions on a QKD implementation. This is exemplified in Device Independent (DI) QKD, which can provide an information theoretic secure key even if the physical quantum devices used in the protocol are not trusted to behave as expected^31, 32^. While progress in the theory has been underway, laboratory experimental demonstrations remain a challenge due to amongst other things the requirement for a loophole free Bell test. Even with experimental progress the secret key rate is anticipated to be extremely low, on the order of 10^−10^ bits per pulse, and only possible over very limited distance^33, 34^.

A more feasible proposal is Measurement Device Independent (MDI) QKD^35, 36^, where the detector units are untrusted but the transmitters must be trusted as in standard QKD. MDI-QKD can remove all of the detector based side-channel attacks but still remains vulnerable to source based attacks. It has been experimentally demonstrated in several recent papers^37–40^, including outside of the laboratory in a field trial environment^41, 42^. However there remain challenges, including the difficulty of synchronising and interfering two phase randomised independent sources separated by large distances, especially at the clock rates used by modern conventional QKD systems. This typically limits the secure key rate to values much lower than conventional QKD in practical scenarios, despite a recent laboratory proof of principle demonstration of high bit rate MDI-QKD^43^. Additionally MDI-QKD uses a three party configuration, which is not as straightforward to integrate into existing communication infrastructure.

Here we focus on providing security against side-channel attacks for conventional QKD, which can work reliably at high key rates alongside existing telecom infrastructure. It is also worth noting security techniques developed for this purpose are also applicable for the transmitter units (Alice and Bob) in MDI-QKD systems, which use a similar architecture and are vulnerable to source side channel attacks. Efforts are currently underway towards the standardisation of QKD^44, 45^, including implementation security and countermeasures against side-channel attacks. As such we aim to develop possible solutions towards the goal of future implementation standards, which are urgently needed to allow for robust testing and certification of security.

In the following section we report a QKD system which has been designed to this end, to provide not only theoretical but also practically implemented security. The section following this reports the system’s installation in a telecom fibre network for field testing, and the performance of a newly developed security proof providing security against more general attacks than usually considered. The Methods section provides additional details about the QKD system hardware, stabilisation, security countermeasures and post processing.

## QKD System

**Figure 1:**
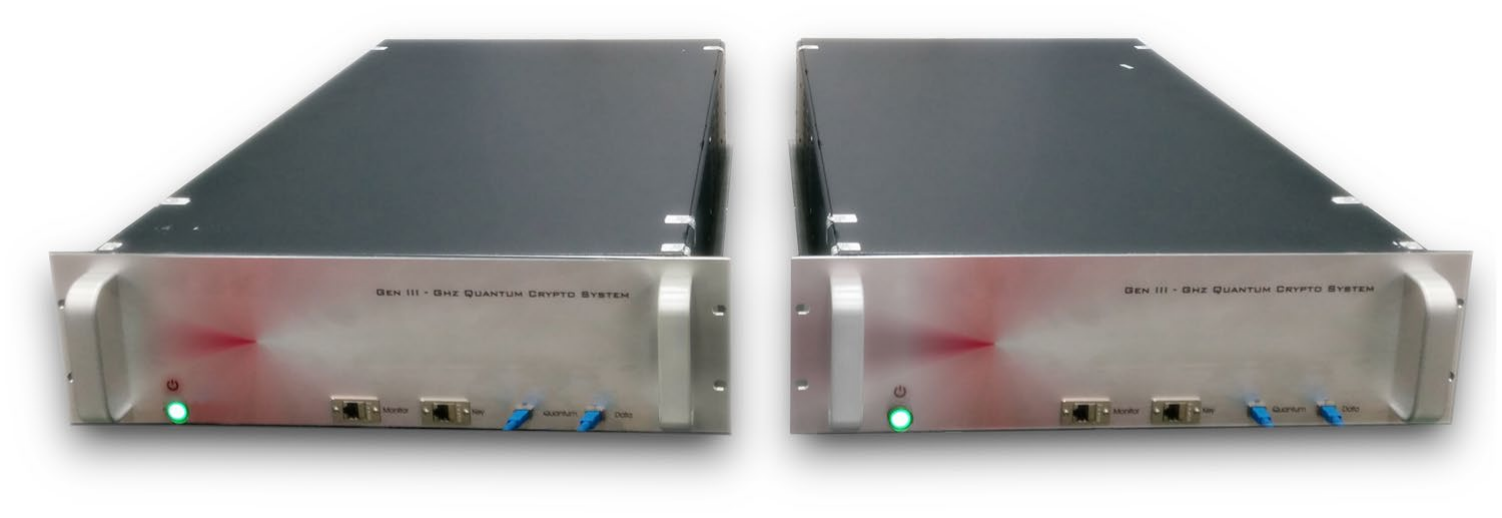
Photograph of the QKD system transmitter and receiver. The units are 19 inch rack sized (3 U high).

**Figure 2:**
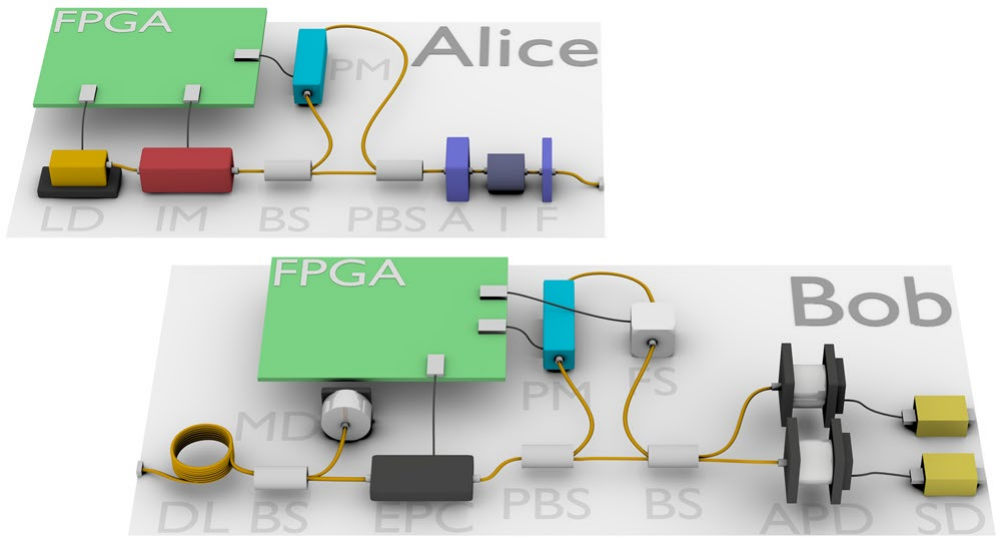
Schematic diagram of main components of the QKD system, showing the transmitter (Alice) and receiver (Bob). LD: Laser diode, IM: Intensity modulator, BS: Beam splitter, PBS: Polarising beam splitter, A: Variable optical attenuator, I: Optical isolator, F: Narrow band pass optical filter, DL: Delay line, MD: Monitoring detector, EPC: Electronic polarisation controller, FS: Fibre stretcher, APD: Avalanche photodiode detector, SD: self-differencing circuit.

The prototype QKD system consists of rack mount server sized (19 inch wide and 3U high) units, as shown in Fig. 1. One unit is the transmitter (“Alice”), and the second unit a receiver (“Bob”). The system is based around the well-known decoy state BB84 protocol and uses phase encoded optical pulses with sub single photon intensities to transmit the quantum information. The QKD system implements an automated initialisation and alignment routine which enables key distribution to begin operating within several minutes of a cold start, with no user input or adjustment required. The system additionally implements component monitoring for both security and reliability, as well as refined stabilisation subsystems to provide consistent operation under harsh operating conditions such as those experienced during transmission through aerial fibre cables. A web browser based graphical user interface is also implemented to allow for user friendly control and monitoring of the system and all security and component subsystems. Figure 2 shows an outline schematic of the major components in the QKD transmitter and receiver, with further details on the standard QKD components provided in the Methods section and ref. 46, and the implementation security features discussed in the following section.

### Implementation Security

As shown in Table 1, there are three typical source side channel attacks for this type of QKD system. Photon number splitting attacks can be well controlled using decoy states, and 3 intensities levels (signal μ ≈ 0.4, decoy μ ≈ 0.1 and vacuum μ ≈ 0.0007 photons/pulse) are implemented in the system for this purpose – see the Methods section for further details. Phase randomisation of subsequent pulses without the need for additional active components from the particular laser source used in the transmitter has been tested^47–49^, thus mitigating this side channel. An alternative approach would be using a small number of discrete random phases to guarantee security^50^. In this case, however, an additional source of random numbers is needed and the approach has only been demonstrated in the infinite-key limit to date. The laser diode temperature and output power is continuously monitored to ensure it is in the correct operating regime for phase randomisation and for QKD, and the system output power is constantly monitored and kept stable using an automated variable optical attenuator. If any anomalies in these quantities are detected QKD is suspended and an alert displayed in the user interface software.

The remaining main source based attack is the Trojan horse (also called large pulse) attack. In this attack an adversary directs intense light into a QKD system and measures the reflected light in order to gain information about the state of the components inside the system, which can leak information on the key. The system’s vulnerability to this type of attack has been analysed and quantified, with full details in ref. 20. This analysis principally considers attacks on the phase modulator, but it has recently been extended to also cover intensity modulator attacks by Tamaki *et al*.^21^. These type of attacks can often be more dangerous, but with the conservative choice of countermeasure components (discussed in the following paragraph and the Methods section) the system satisfies the security requirements for both phase modulator and intensity modulator. The analysis is based on characterising the reflectivity of components inside the transmitter (typically around 40 dB) and the maximum amount of input light possible before destructive fibre damage occurs (typically around 5 W). Based on these values a bound can be placed on the maximum amount of reflected light it is possible for a malicious eavesdropper to collect, and this can then be used to bound the information gain possible through the attack. This information gain is incorporated into the secure rate calculation, and privacy amplification used to remove it as normal for leaked information.

Additional optical components can be added to the transmitter to reduce the amount of reflected light, and reduce the information gain and required extra privacy amplification to an arbitrarily small amount. These optical components are show in Fig. 2: Attenuators (A), which provide equal attenuation in both directions of light travel; isolators (I), which attenuate strongly in only one direction; and narrow band pass wavelength filters (F), which provide strong attenuation outside a small wavelength window. The use of wavelength filters is important to prevent attacks exploiting possibly increased reflectivity of components and decreased attenuation outside of the usual 1550 nm operating wavelength of the system^51^. Further details on these components is provided in the Methods section.

The receiver unit is protected from Trojan horse attacks against the phase modulator through the use of an appropriate optical delay line (DL in Fig. 2) combined with the GHz modulation clock rate. Due to the photons travel time through the delay line and the basis modulator switching time this makes it impossible for Eve to receive any back reflected light from the phase modulator before the modulated photon has been detected by Bob^52^. The only information Eve can gain from Bob’s modulator is on the basis measured, and this information is of no use after the detection has taken place (at which points the basis is publically revealed).

To provide a basic guard against potential APD blinding attacks the input optical power is monitored at the receiver as shown in Fig. 2, with a beam splitter (BS) and optical power monitor (MD) located directly after the receiver’s input port from the transmission fibre. Approximately 99% of the input is directed to Bob’s interferometer as usual, with 1% directed to the optical power monitor. In addition the APD module’s temperature is continuously monitored for any anomalies, which will further constrain possible hacking attacks^22^. Any out of range discrepancies in light input or temperature cause QKD to be suspended and an alarm to be raised in the user interface. These countermeasures restrict the range of feasible blinding attacks, but a tight connection with a security proof and testable assumptions is still lacking. Therefore, they cannot be considered a complete solution. For instance the presented technique has limitations due to the low response of the monitoring detector to ultra-narrow optical pulses.

### Active Stabilisation

Due to fluctuations in environmental conditions affecting both the transmission fibre and the QKD units there are several time varying noise sources which affect the system; these must be continuously compensated for to maintain stable key distribution operation – details of these stabilisation systems are provided in the Methods section.

During field trials, and in practical use cases, the transmitter and receiver QKD units will be placed in separate and remote locations and will operate over fibres which may be exposed to uncontrollable environmental perturbations. To enable the system to operate at high key rates within these potentially rapidly changing conditions more specialised stabilisation algorithms have been developed. The algorithm employed is based on Proportional Integral Differential (PID) control, and provides an output signal influenced by both the current and feedback signal history and its expected value. This algorithm is used both for stabilising the interferometer and the polarisation drift in the fibre.

**Figure 3:**
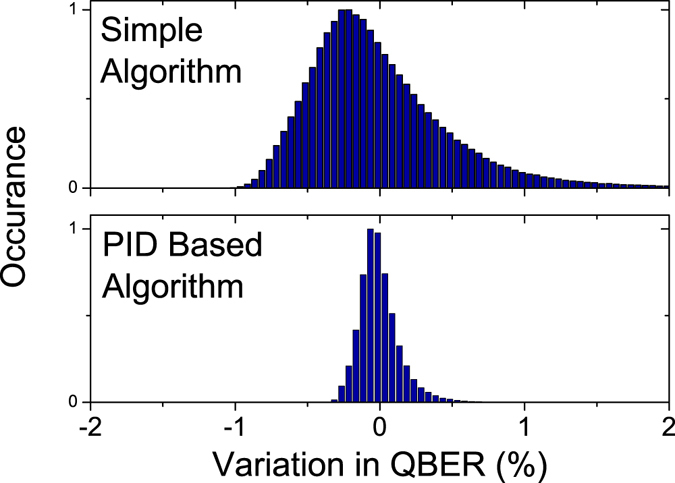
Histogram of QBER variation from the mean over time, using the newly developed PID based stabilisation algorithm (lower) as compared to the previous simple algorithm (top).

A comparison between the newly developed specialised algorithm and a simpler fixed rate algorithm (used for example in the QKD system described in ref. 46) is shown in Fig. 3. As can be seen from the figure the variation in QBER is much reduced with the PID based algorithms.

## Results

**Figure 4:**
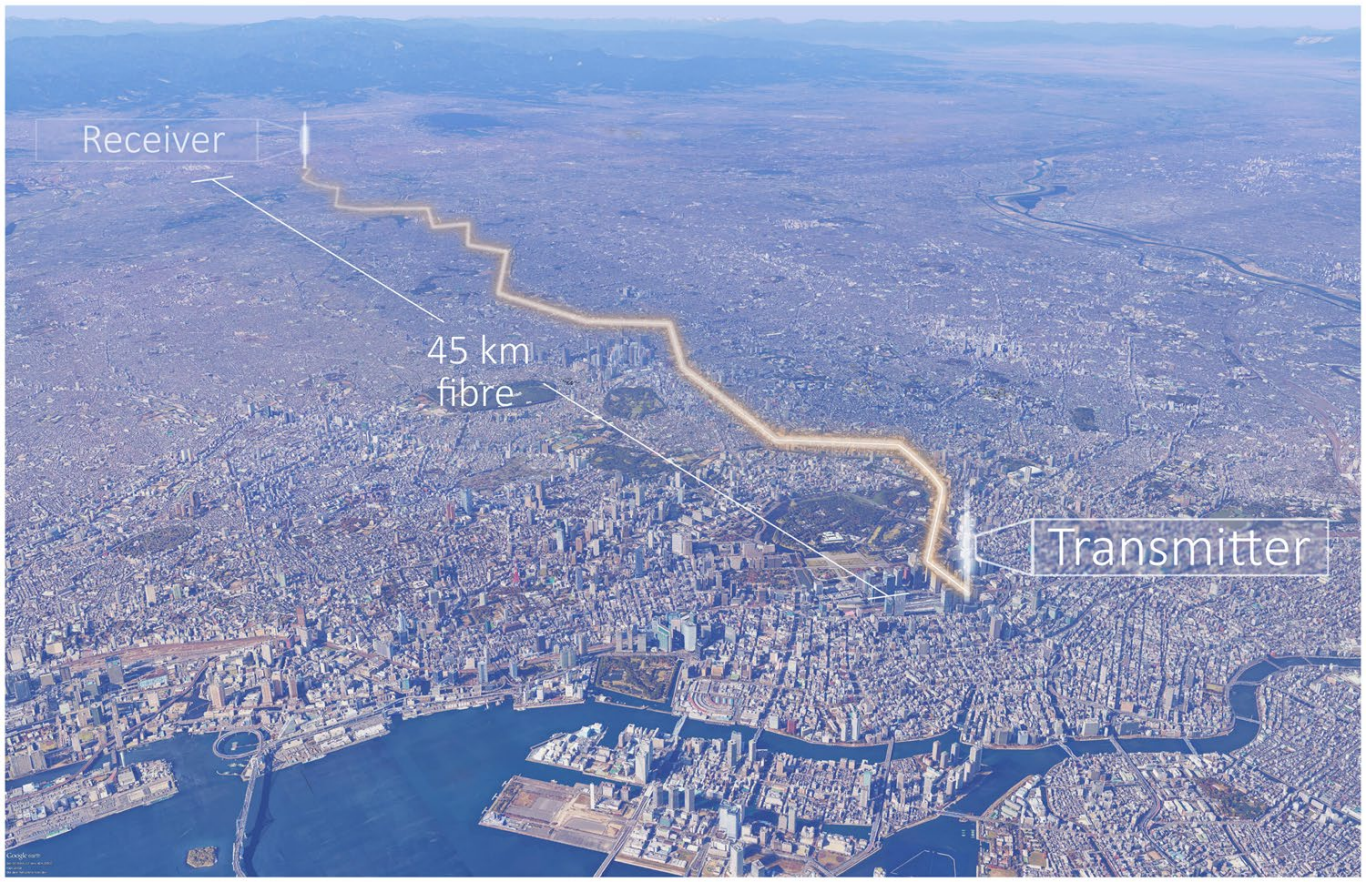
Location of the field trial of the QKD system, with the transmitter sited in central Tokyo and the receiver towards the western edge of the city. The two locations are connected by an installed telecom fibre pair with a length of 45 km and loss of 14.5 dB. Map data courtesy of: Google Earth, SIO, NOAA, U.S. Navy, NGA, GEBCO, Image Landsat and Japan Hydrographic Association.

The QKD system described in the previous section was installed into a metropolitan area fibre telecom network^53^ as shown in Fig. 4. A fibre optic cable of 45 km length connects an office building in central Tokyo to a location in the western suburbs of the city. The transmitter is installed in a server rack at the central location and connected to the receiver in the western location by two fibres from the cable; one is used for quantum signals and the second for all other communication data, such that no external network connection is required for the QKD system to operate. The fibre is of standard SMF-28 type with a total characterised loss of 14.5 dB, equivalent to 0.33 dB/km – this is increased compared to the typical laboratory fibre loss of 0.2 dB/km mainly due to splice and other connector losses. Approximately half of the fibre is located in underground ducts and half suspended above ground on aerial poles. Aerial fibre is in general much more exposed to environmental changes such as temperature and wind induced movement, which can affect the transmission characteristics (for example transit time and birefringence).

**Figure 5:**
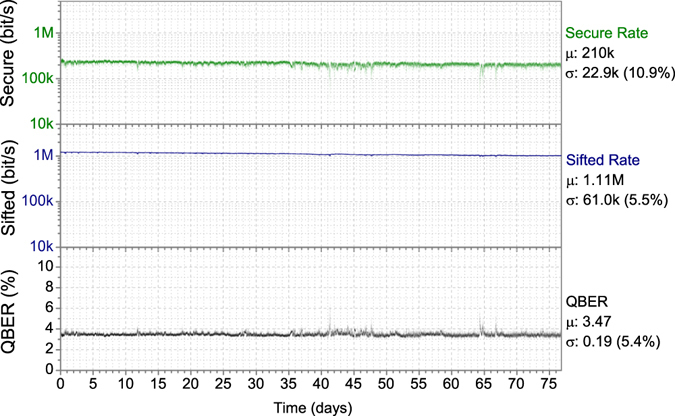
Field trial performance of the QKD system installed in a 45 km telecom fibre link over a 77 day period, with collective attack security. From upper to lower the secure key rate, sifted key rate and QBER are shown along with their mean value (μ) and standard deviation (σ).

Following installation the system operated continuously for several extended periods of time, during which the system was entirely automated with no user control or adjustments made to the system. Results from a typical 77 days of continuous operation are shown in Fig. 5 (the field trial continuous operation duration was limited by external factors such as transmission fibre maintenance or power outages, with uninterrupted fibre access longer term continuous operation would be possible).

**Figure 6:**
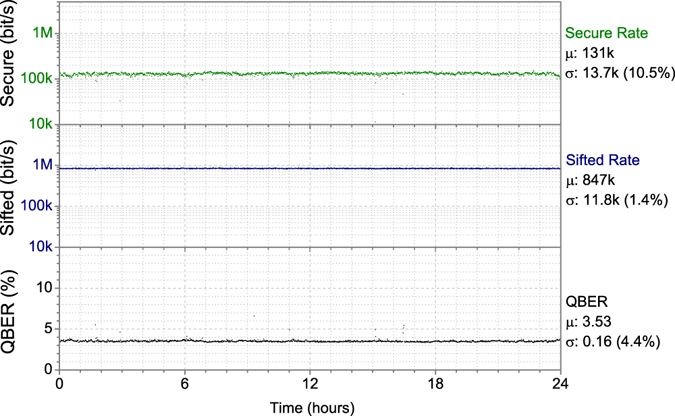
Field trial performance of the QKD system installed in a 45 km telecom fibre link, with general attack security. From upper to lower the secure key rate, sifted key rate and QBER are shown along with their mean value.

Following the extended field trial reported in the previous section the system was upgraded to use a newly developed version of the security proof which provides security against more general attacks^54^. Results from operation over the same 45 km of installed fibre are shown in Fig. 6, which to our knowledge is the first QKD field trial guaranteeing theoretical security against a class of attacks wider than collective attacks, including finite-key size effects and decoy states.

## Discussion

The system performance, deployed over the 45 km installed fibre, is shown in Fig. 5 over a period of 2.5 months (the operation time limited by external power supply and transmission fibre maintenance). The sifted key rate (94% of the raw rate) averaged 1.11 Mbit/s and QBER 3.47%. Both remained stable over the period with ≈5% standard deviation, due to newly developed active stabilisation feedback subsystems able to cope with variable weather conditions. During this time the secure key rate averaged 210 kbit/s and in total 1.33 terabits of secure key data was distributed. Despite several security enhancements to the current system the secure key rate is similar to the rate during a shorter field trial of a previous system^46^ while the variation of all parameters is reduced, mainly due to the improved stabilisation systems. The secure key rate is calculated with composable security (failure probability ε = 10^−10^) against collective attacks^55^ on finite key block sizes (50 Mbit), with error correction and privacy amplification performed on this block in real time – further details are described in the Methods section.

The system additionally implemented a newly developed security proof, aimed at covering a more general class of theoretical attacks, and with this over a 24 hour field trial period averaged a secure key rate of 131 kbit/s (Fig. 6). The key rate is reduced compared to collective attacks only (Fig. 5), despite the QBER being approximately the same. This reduction is partially due to the more general attacks considered and partially to the reduced sifted key rate, caused by the optimal value of the majority basis fraction (the photons used for the final key) being smaller for the general attacks case.

We have reported the development and field trial performance of a high speed QKD system. The system implements security countermeasures to prevent against side-channel hacking attacks, in particular against Trojan horse attacks, as well as phase randomisation, photon number splitting and detector blinding attacks. Additionally components of the system including the laser diode and APDs are monitored continuously. We believe that testable implementation security countermeasures in conjunction with privacy amplification will be a useful tool for future QKD systems (including MDI QKD which requires countermeasures for the Alice and Bob units), and will help QKD to maintain robust security guarantees even in the presence of non-ideal realistic components.

## Methods

### QKD System details

The system is based around FPGAs and integrated electronics. It operates at a 1 GHz transmission clock rate, with a 1550 nm distributed feedback laser (LD) in the transmitter unit producing photon pulses which are subsequently attenuated to contain approximately 0.4 photons per pulse on average. Decoy states^56, 57^ are implemented using an intensity modulator (IM) to allow for a high secure key rate secure against possible photon number splitting attacks, with ~1% of pulses transmitted with a reduced photon flux of 0.1 photons per pulse and <1% as a vacuum pulse containing 0.0007 photons per pulse. The intensity in the vacuum pulses is limited by the extinction ratio of the intensity modulator used for the state preparation.

All photon states pass through an asymmetrical Mach-Zehnder interferometer in the transmitter, one arm of which contains a phase modulator (PM) to encode four discrete phase values (2 basis each with 2 states) onto the photon pulse. Asymmetrical, or efficient, BB84 basis selection probabilities^58^ are used to increase the secure key rate, with the majority basis selected with 97% probability at both the transmitter and receiver. The decoy fractions, photon fluxes, and basis probabilities are all optimised through simulation to produce optimally high secure key rates. A fibre Bragg grating is also employed to reduce the effects of chromatic dispersion during transmission as the system is designed to be used over standard telecom fibre where chromatic dispersion can cause QBER degradation^59^.

A matched Mach-Zehnder interferometer in the receiver decodes the photon’s phase into output detector path information, using a phase modulator (PM) in one arm for active QKD basis selection. The interferometer pair is constructed using polarisation dependent beam splitters (PBS), to ensure photons travel through opposite paths in the interferometer pair (long-short or short-long) and thus always arrive at the final beam splitter coincidently. An electronic polarisation controller (EPC) is placed before the interferometer to compensate polarisation rotations in the transmission fibre, and an electrically driven fibre stretcher (FS) in one arm of the interferometer compensates path length changes.

Following the interferometer, single photon detection is performed using self differenced^60^ InGaAs avalanche photodiodes (APDs) thermoelectrically cooled to −30 °C. The self differencing (SD) circuit allows the APDs to be gated in Geiger mode at 1 GHz without the excessive noise which would normally result at such gating rates. The detectors operate at an efficiency of 20% with a 4% afterpulse probability and 2 × 10^−5^ dark counts per gate.

### Trojan horse components

**Figure 7:**
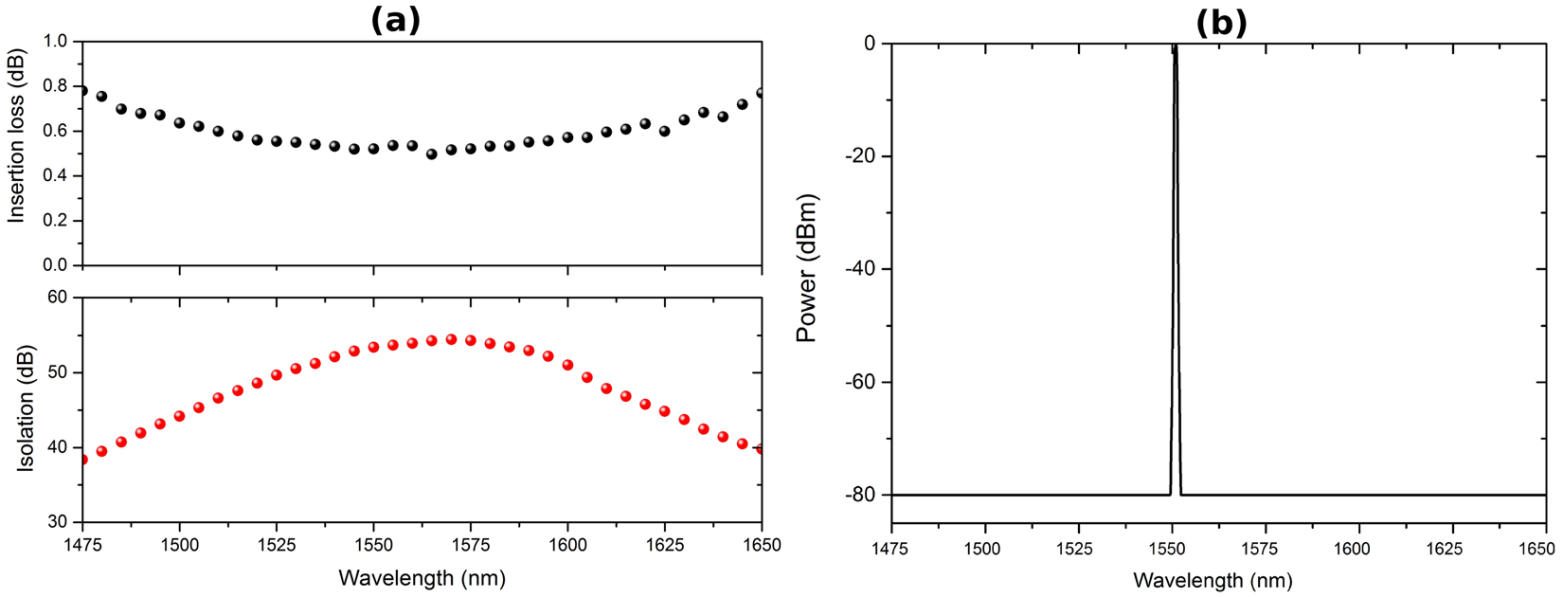
Optical performance of (**a**) isolators and (**b**) wavelength filters, used to provide protection against Trojan horse attacks.

Figure 7 shows typical optical properties of two of these components, an isolator in (a) and wavelength filter in (b). The isolators used typically provide in excess of 60 dB of attenuation in one direction and less than 0.6 dB in the reverse case. The wavelength filter provides close to no loss at its central wavelength and approximately 80 dB of loss outside of this. By combining a small number of optical components – a 40 dB attenuator, 60 dB isolator and wavelength filter – sufficient total round trip attenuation can be achieved (on the order of 200 dB including component reflectivity) to make the possible information leakage from Trojan horse attacks negligible^21^.

### Stabilisation systems

There are three main stabilisation systems which operate continuously to maintain the raw key rate and QBER at their optimum values.

Firstly the path length of the fibre optic based interferometers in the transmitter and receiver change with temperature, and so to compensate for this change and maintain identical path lengths as required an electrically driven fibre length stretching component is placed in the receiver’s interferometer. This component is driven continuously based on a feedback signal provided by higher intensity optical stabilisation pulses which are sent in a known phase state and randomly replace quantum pulses a small fraction of the time.

The second main stabilisation subsystem is required to compensate the polarisation rotation which occurs to photons during travel through the transmission fibre. While the quantum information is encoded on the photon’s phase, polarisation is used to increase the efficiency of the interferometer pair by avoiding paths where photons travel through both long arms or both short arms of the interferometers (these cases would not interfere and fall outside of the detector gate period, reducing the detected photon count rate). As the transmission fibre is subject to environmental movement and expansion the birefringence changes and so the output polarisation rotates constantly, and to compensate for this an electronic polarisation controller (EPC) is employed before the interferometer in the receiver. This EPC is driven continuously based on a feedback signal provided by the detectors’ count rate.

The third main stabilisation subsystem is related to the photon travel time variation during transmission through the fibre, caused primarily by fibre expansion and contraction due to environmental temperature changes. Based on the detected photon count rate the clock delay in the receiver is adjusted so that the photon arrival time always matches the centre of the detectors’ gate period and the centre of the phase modulator period.

### QKD Post-processing

The secure key is produced from a finite sized raw key, and as such all estimated quantities used in the secure key size calculation are subject to statistical bounds. In order to obtain the highest secure key rate tight bounds are required, and this requires larger raw key block sizes to be used during the post processing phase, in particular for privacy amplification. Privacy amplification using the simple matrix multiplication approach traditionally employed scales as *N* ^*2*^ in computational complexity with increasing block size *N*. As such it becomes infeasible to use with the large block sizes required for high key rates. Instead we implement a number theoretic transform based algorithm which scales almost linearly (*N* log (*N*)) with block size^61^. This enables block sizes of greater than 50 Mbit to be privacy amplified in real time even at Mbit/s key rates.

Error correction is implemented using the Cascade algorithm^62^, with typical error correction efficiencies of around 15% above the theoretical minimum (f = 1.15). While LDPC based error correction^63^ has also been investigated^64^ and found to have a somewhat improved efficiency (f ≈ 1.10), in practice the overall increase in secure key rate has been found to be small (on the order of 1%) once increased block failure rates are taken into account. Cascade (or LDPC) based error correction runs in real time at the Mbit/s raw key rates generated by the system.
